# Effects of asparaginases and L-carnitine on Western-diet-induced hepatosteatosis in mice

**DOI:** 10.12688/f1000research.75870.1

**Published:** 2022-02-01

**Authors:** Mona Ali Mahmoud Assar, Martina Hüffel, Mamdouh Afify, Ralf Weiskirchen, Albrecht Eisert, Rene Tolba, Julia Steitz

**Affiliations:** 1 Institute for Laboratory Animal Science, Faculty of Medicine, RWTH Aachen University., Aachen, Nordrhein Westfalen, 52074, Germany; 2 Department of Zoology, Faculty of Science, Menoufia University., Shibin Elkom City, Egypt; 3 Department of Pathology, Faculty of Veterinary Medicine, Cairo University, Cairo, Egypt; 4 Clinic for Cardiology, Angiology and Internal Intensive Medicine Pneumology (Medical Clinic I), Faculty of Medicine, RWTH Aachen University, Aachen, Nordrhein Westfalen, 52074, Germany; 5 Institute of Molecular Pathobiochemistry, Experimental Gene Therapy and Clinical Chemistry, Faculty of Medicine, RWTH Aachen University, Aachen, Nordrhein Westfalen, 52074, Germany; 6 Institute of Clinical Pharmacology, Faculty of Medicine, RWTH Aachen University, Aachen, Germany; 7 Hospital Pharmacy, University Hospital RWTH Aachen, Aachen, Germany

**Keywords:** asparaginases, hepatosteatosis, L-carnitine, hepatotoxicity.

## Abstract

Abstract

**Background: **Asparaginases are common chemotherapeutic agents used for the treatment of acute lymphoblastic leukemia as a single or combinational therapy. Accompanying hepatotoxicity makes its use in elderly patients with pre-conditions, as obesity or other hepatopathies, difficult. Various hepatoprotective compounds like, L-carnitine, are discussed to ameliorate the induced hepatotoxicity.

**Methods:** Here we aimed to establish a mouse model to study the effect of asparaginases (L-asparaginase and Oncaspar) and L-carnitine on Western-diet-induced hepatosteatosis in mice. Dose-escalation studies were performed to analyze asparaginases induced hepatotoxicity in C57BL/6 mice with normal or fatty livers. Subsequently, the effect of L-carnitine to improve the induced toxicity was tested.

**Results:** Our results showed mild-to-moderate hepatotoxic effects while the Western-diet induced a higher degree of vacuolization and hepatocyte damage in liver tissue. Testing of L-carnitine in the established models did not show any protective effect on the toxicity or impairment of the efficacy of asparaginases.

**Conclusion:** The here established models were able to demonstrate the asparaginase-induced hepatotoxic effects which were enhanced by the Western-diet. However, to test potential ameliorating drugs, the models might need some improvements.

## Introduction

The bacterial-derived enzyme asparaginase has been successfully used in chemotherapeutic regimens developed for the treatment of acute lymphocytic leukemia (ALL) in children and adult patients. ^1^^,^^2^ Clinically, three common forms of asparaginase are in use: a native L-asparaginase isolated from *Escherichia coli*, a pegylated form (polyethylene glycol asparaginase, also called pegaspargase or Oncaspar) and an *Erwinia chrysanthemi* derived asparaginase known as *Erwinia* asparaginase (Erwinase). ^2^ Pegaspargase has been developed to reduce the immunogenicity of the enzyme and prolong its half-life in plasma. ^3^

Despite the essential role of L-asparaginase as a chemotherapeutic agent, adverse effects have been reported and comprise hypersensitivity reactions, liver injury, pancreatic toxicity, coagulopathy, immune suppression and effects on the central nervous system resulting in the decrease of L-asparagine and L-glutamine levels. ^4^

Even though asparaginases are efficiently applied as combinational chemotherapeutic agents in children’s and adults’ ALL, these drugs can induce side effects mainly in elderly patients with pre-existing conditions such as hepatopathies. ^5^ With aging, people often become overweight and obese increasing the risk of additional health problems and co-morbidities. ^5^ Previous studies have reported that patients with preceding hepatic steatosis or hepatitis, developed more severe side effects after treatment with chemotherapeutic drugs ^6^^,^^7^ or showed even fatal liver failure. ^8^ Various compounds *e.g.*, L-carnitine (L-C) have been described to ameliorate liver damage in patients with non-alcoholic fatty liver disease. ^9^^–^^11^ Additionally, L-C reduced kidney and heart toxicity triggered by cisplatin and doxorubicin chemotherapeutic drugs. ^12^^,^^13^

We aimed in our experiments to establish a fatty liver model in mice to study the effects of asparaginases on the liver and the potential intervention with L-C to ameliorate toxicity.

## Methods

### Ethical approval

Animal studies were approved by the Governmental Animal Care and Use Committee at the LANUV NRW, Recklinghausen, Germany (approval number: AZ 87-51.04.2010.A278 and AZ 84-02.04.2016.A433), and performed in accordance with the German legislation governing animal studies following the guide for the care and use of Laboratory Animals (NIH publication, 8th edition, 2011) and the Directive 2010/63/EU on the protection of animals used for scientific purposes. Our animal study was conducted according to the ARRIVE guidelines 2.0 using the ARRIVE Essential 10 checklist for pre-clinical animal studies.

### Animal models

**Figure 1.**
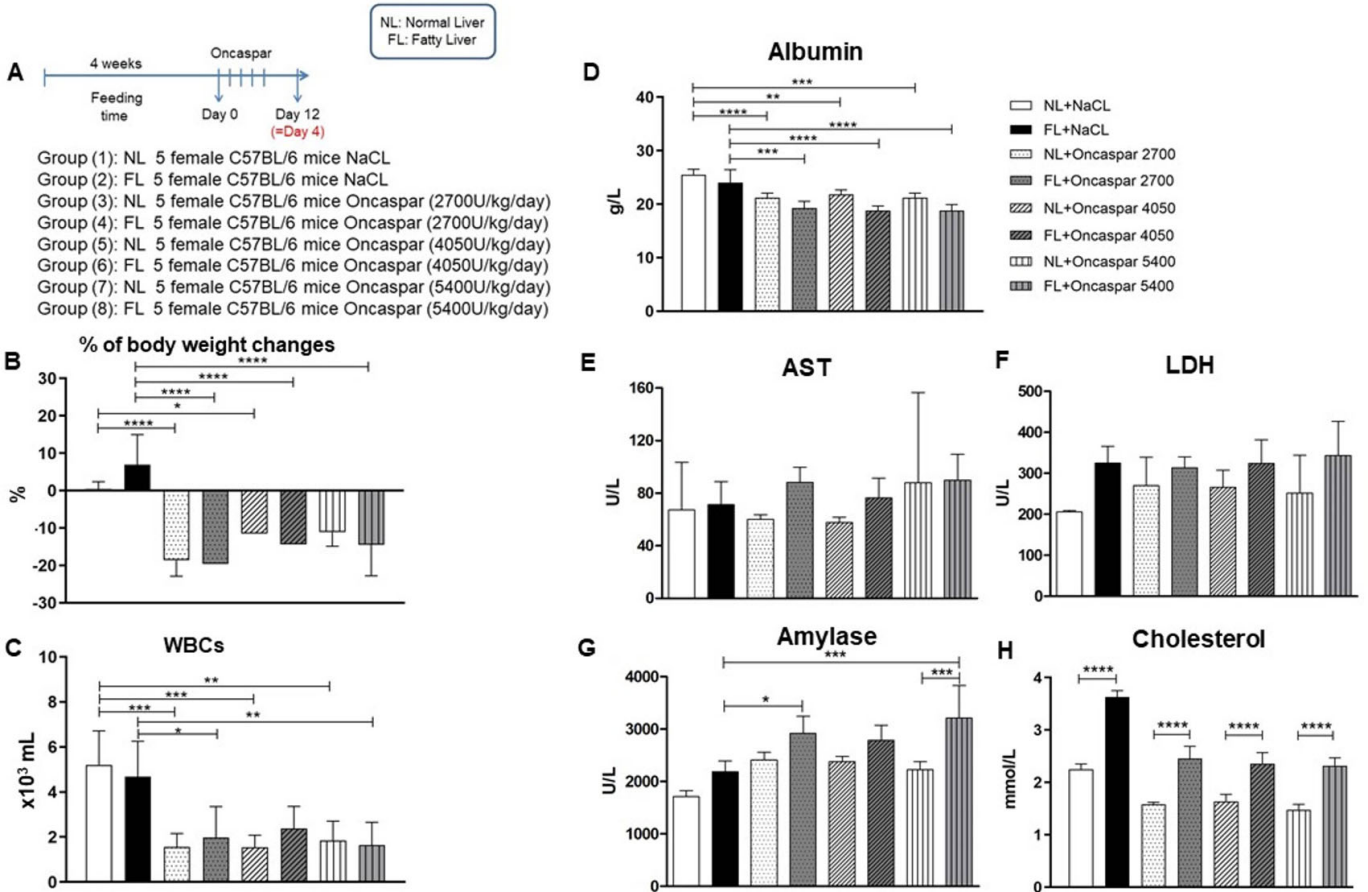
Experimental design (A), % bodyweight changes (B), WBC (C), Albumin (D), AST (E), LDH (F), Amylase (G), Cholesterol (H) are shown as means±SD at the 4-day time point. * *p* < 0.05, ** *p* < 0.01, *** *p* < 0.001, **** *p*< 0.0001.

6-week-old female and, 3–5 weeks old male C57BL **/**6NCrl mice were obtained from Charles River, Sulzfeld, Germany. Our best efforts were made to minimize suffering and pain of animals by applying 3Rs principle in our animal experiments. Water and diet were supplied *ad libitum* and animals were housed under specific-pathogen-free condition according to the Federation of European Laboratory Animal Science Associations (FELASA) guidelines. Mice were housed in a barrier housing facility at the Institute for Laboratory Animal Science in Aachen, Germany. The room temperature was 21–23 °C with a humidity of 30 –70 %. The light intensity was below 200 lux at a height of 1 m and a light-dark rhythm of 12 hours each. Animals were inspected on daily basis. The animals were kept (max. 5 mice/cage) in type II L cages according to the requirements of Annex 3 of the EU Directive 2010/63. A total of 40 female mice were utilized for dose-escalation study with Oncaspar ^®^ (Sigma-Tau, Rome, Italy) and fed for the first 4 weeks with either normal (normal liver, NL) or Western-diet (fatty liver, FL) (Sniff Spezialdiäten GmbH, Soest, Germany). After feeding duration, animals were randomly divided into eight groups (n=5). Five animals per group received 5 intravenous injections of Oncaspar ^®^ with either 2700 U/kg, 4050 U/kg, and 5400 U/kg bodyweight into the lateral tail vein in a maximum volume of 5 mL/kg body weight every two days. Control groups received corresponding volumes of 0.9% NaCl solution ( Figure 1A).

Analysis of L-asparaginase (Spectrila ^®^, Medac, Wedel, Germany) was performed in male mice after 8 weeks of Western-diet or normal diet. A total number of 120 animals were randomly divided into 6 groups (n=20). Twenty animals/group received 5 doses of 5400 U/kg bodyweight L-asparaginase or 0.9% NaCl solution as control every two days ( Figure 3A). In indicated groups, animals received L-C (300 mg/kg bodyweight) intraperitoneally.

Bodyweight and health conditions were measured daily during the treatment period up to day 4 or day 28 after the last treatment. A modified version of the scoring system described by Morton and Griffiths ^14^ was utilized twice a week during feeding duration and daily during injection duration for assessment of severity scoring. Animals were anesthetized for final procedure with inhalation anaesthesia using isoflurane. The maximum amount of blood was collected from retro-orbital sinus under isoflurane anaesthesia using capillary tubes and finally sacrificed with an overdose of isoflurane and organs were collected for further analysis.

### Calculation of bodyweight changes

Bodyweight changes were calculated based on the bodyweight of the animal at day 0 (before treatment start) and the necropsy time points (days 4 and 28) and were expressed in percentage.

### Blood/serum analysis

White blood cell counts (WBCs) were measured in K-EDTA blood received from the final retro-bulbar blood draw using the Celltac α MEK-6550K (Nihon Kohden Europe GmbH, Rosbach vor der Höhe, Germany) analyzer. Indicated serum parameters were analyzed in serum using the VITROS 350 (Ortho Clinical Diagnostics, Neckargemünd, Germany) system.

### Histopathological analysis

**Hematoxylin and eosin staining**

Liver samples were fixed in 4% neutral buffered formaldehyde (Carl Roth GmbH, Karlsruhe, Germany) for 48 h, dehydrated and embedded in paraffin. 4–6 μm thick liver sections were stained with hematoxylin and eosin (H&E). Grading of liver damage was performed by a board-certified pathologist in a blinded manner in five randomly selected fields in each liver specimen at 400× magnification. A scale of 1–5 was utilized for grading of lesions; 1=no or negligible changes, lesions affecting 0–10% of the field; 2=mild, lesions affecting 10–30% of the field; 3=moderate, lesions affecting 30–60% of the field; 4=moderate to severe, lesions affecting 60–80% of the field; and 5=severe, lesions affecting >80% of the field. Different histopathological changes were examined: hepatocytic vacuolization, degenerative changes, congestion, cellular infiltration, and Kupffer cell activation.

**Oil Red O stain**

Standard Oil Red O (ORO) stain (Merck, Darmstadt, Germany) was used for the detection of neutral triglycerides and lipids in 5 μm cryo-sections of the liver embedded in Tissue-Tek (Sakura Finetek, Torrance, CA, USA). ImageJ software (ImageJ, RRID:SCR_003070) was utilized for quantification of accumulated lipids. Five bright-field images were captured per slide using a light microscope at 200× magnification.

### Statistical analysis

Statistical analysis was performed using GraphPad Prism 8 software (RRID:SCR_002798 ) (GraphPad Software Inc., San Diego, CA, USA) and results are expressed as Mean ±SD. One-way analysis of variance (ANOVA) for multiple comparisons between the mean of different treatment groups and Bonferroni correction test was used. Results were considered significant for *p*-values <0.05.

## Results

First, a dose-escalation study with Oncaspar was performed to evaluate the hepatotoxic effect after five intravenous injections in normal (normal liver) or Western-diet (fatty liver) fed female mice ( Figure 1A).

Western-diet feeding for 4 weeks resulted in a not significant elevation of bodyweight changes compared to the control group fed with a normal diet. All doses of Oncaspar induced a marked reduction in bodyweight changes in all groups compared to their controls ( Figure 1B). To evaluate the efficiency of the chemotherapeutic drug Oncaspar, WBC were measured showing significant reduction in all Oncaspar treatment groups (except for FL+ Oncaspar 4050) compared to their control groups ( Figure 1C).

For the evaluation of toxicity, clinical chemistry parameters in serum were analyzed and are shown in Figure 1 and Table 1. Significant reduction in serum albumin (ALB), as a marker of hepatic protein synthesis, is observed in all Oncaspar treatment groups ( Figure 1D) and similar results were observed for total protein (TP) ( Table 1). Parameters for liver toxicity showed no significant elevations in aspartate aminotransferase (AST) and lactate dehydrogenase (LDH) levels due to the Western-diet. Oncaspar itself did not influence the levels of AST and LDH compared to their control groups ( Figure 1E and F). However, Oncaspar induced a significant elevation in serum amylase, a parameter indicating also pancreatic toxicity, in the FL treatment groups ( Figure 1G). The efficient induction of a fatty liver in mice (FL group) was verified also based on the significant increase of serum cholesterol ( Figure 1H). On the other hand, Oncaspar treatment induced a non-significant decrease in cholesterol in both NL and FL animal groups when compared to their non-treated controls.

**Table 1.** Results of clinical chemistry measurements in sera of female mice with 3 different doses of Oncaspar.

Parameters	NL NaCL	FL NaCL	NL Oncaspar 2700	FL Oncaspar 2700	NL Oncaspar 4050	FL Oncaspar 4050	NL Oncaspar 5400	FL Oncaspar 5400
**Markers of liver toxicity:**								
**ALT (U/L)**	54.25±18.55	54.25±12.71	47.80±3.96	61.25±14.55	53.20±8.76	54.00±7.27	53.80±15.06	59.40±16.13
**ALP (U/L)**	142.00±7.62	120.50±7.77	112.40±10.09 ^⁎⁎^	158.80±15.17 ^†††^	126.40±5.86	136.60±12.12	122.40±6.69	140.00±10.37
**TBIL (mmol/L)**	6.20±5.31	5.60±4.93	6.20±1.30	5.40±4.51	5.60±1.52	5.00±2.65	11.40±2.30	8.20±1.64
**TP (g/dL)**	4.92±0.19	4.83±0.40	4.24±0.17 ^⁎⁎⁎^	4.10±0.24 ^†††^	4.18±0.15 ^⁎⁎⁎^	3.90±0.17 ^††††^	4.10±0.17 ^⁎⁎⁎⁎^	3.84±0.17 ^††††^
**Markers of kidney toxicity:**								
**CK (U/L)**	336.33(391.59)	136.00±60.85	161.40±72.06	200±87.73	62.50±16.78	108.50±36.06	96.33±19.66	330.60±163.63
**BUN (mmol/L)**	6.54±0.77	7.95±1.44	10.14±0.68 ^⁎⁎⁎⁎^	7.36±1.06	9.32±0.99 ^⁎⁎^	10.00±1.15	9.70±1.01 ^⁎⁎⁎^	7.10±0.72
**CREA (mmol/L)**	17.20±2.95	16.00±1.83	22.80±0.84 ^⁎⁎⁎^	19.00±2.55	18.00±1.00	19.60±2.51	20.20±1.92	18.60±1.14
**Markers of pancreatic toxicity:**								
**Lipase (U/L)**	957.50±10.61	892.50±2.12	781.00±30.70	603.00±12.90 ^††^	780.20±129.33	557.33±54.37 ^†††^	680.25±54.89 ^⁎⁎^	630.40±94.23 ^††^
**Other serum parameters:**								
**Glucose (mmol/L)**	10.84±1.68	12.80±3.51	7.45±1.48	7.75±2.20 ^†^	8.65±1.82	9.88±1.56	9.20±1.42	9.28±1.32
**Triglycerides (mmol/L)**	0.91±0.23	1.43±0.50	1.35±0.32	1.14±0.46	1.25±0.17	1.61±0.16 ^⁎⁎^	1.58±0.15 ^⁎⁎^	0.97±0.11

Alanine Aminotransferase (ALT), Alkaline Phosphatase (ALP), Total bilirubin (TBIL), Total protein (TP), Creatine kinase (CK), Blood urea nitrogen (BUN), Creatinine (CREA). ^⁎^ Significant compared to NL NaCl group. ^†^ Significant compared to FL NaCl group. Means±SD. NL= normal liver, FL= fatty liver. ^**^, ^††^p < 0.01, ^***^, ^†††^ p < 0.001, ^****^, ^††††^p < 0.0001.

**Figure 2.**
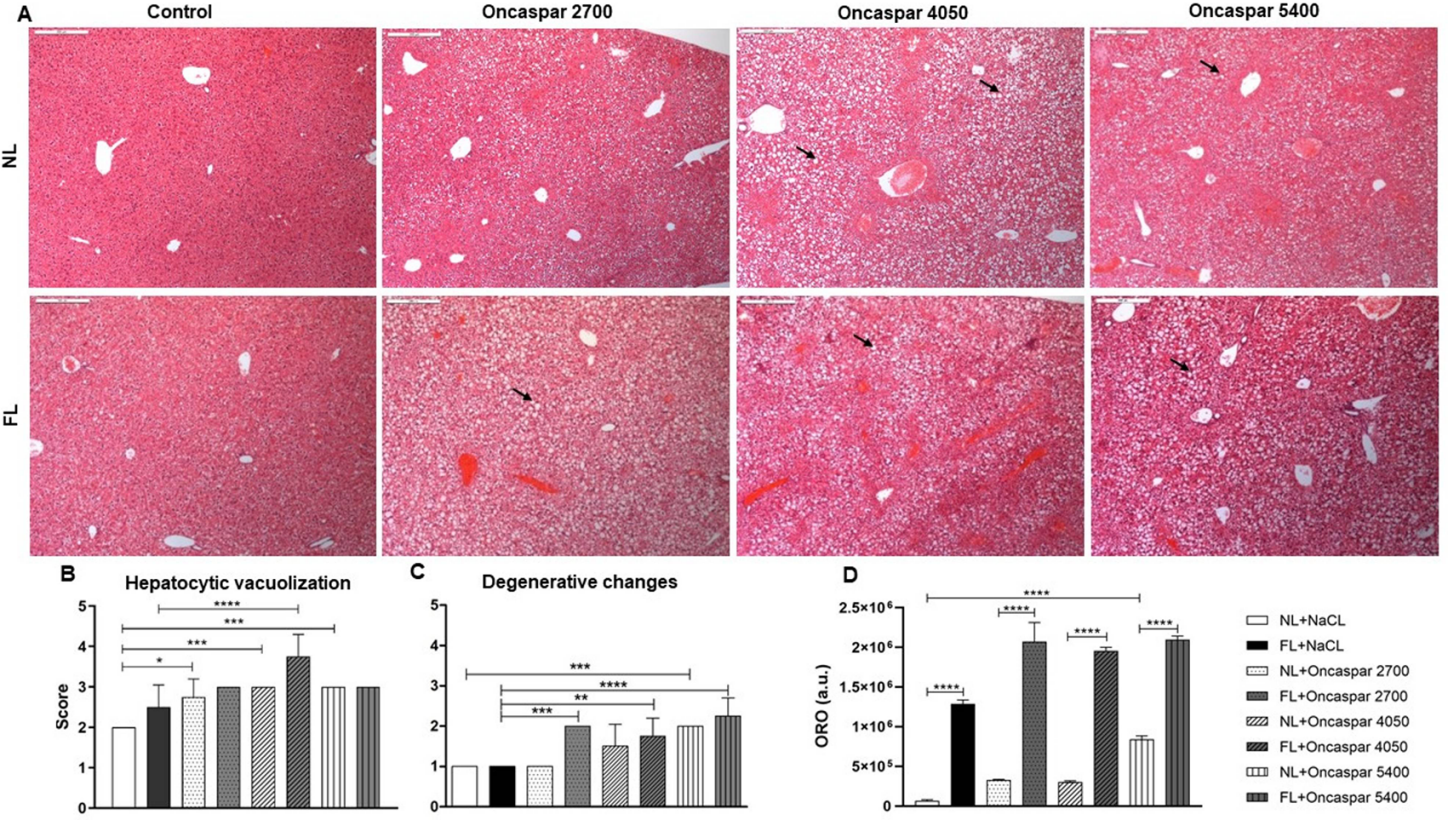
Representative pictures of H&E-stained liver sections at the 4-day time point (100×). Arrows refer to hepatocytic vacuolization (A). Evaluation of hepatocytic vacuolization (B), degenerative changes (C) and Oil Red O (ORO) staining (D) of liver sections are shown as means± SD. Arbitrary units (a.u.). * *p* < 0.05, ** *p* < 0.01, *** *p* < 0.001, **** *p*< 0.0001.

For characterization of the degree of liver damage induced by various doses of Oncaspar as well as Western-diet, histopathological analyses were performed *via* scoring of the pathologic alteration in liver tissue samples. Here, higher levels of degenerative changes in hepatocytes as well as hepatocytic vacuolization were observed in livers after Oncaspar administration ( Figure 2) and were even more pronounced and significant in the fatty liver groups ( Figure 2C).

In addition, the semi-quantitative evaluation of ORO stained cryo-sections visualizing the amount of accumulated lipids in hepatocytes which showed significant accumulation of lipid droplets in hepatocytes cytoplasm in all FL animal groups, while only little lipid accumulation was observed in livers of the NL groups ( Figure 2D).

**Figure 3.**
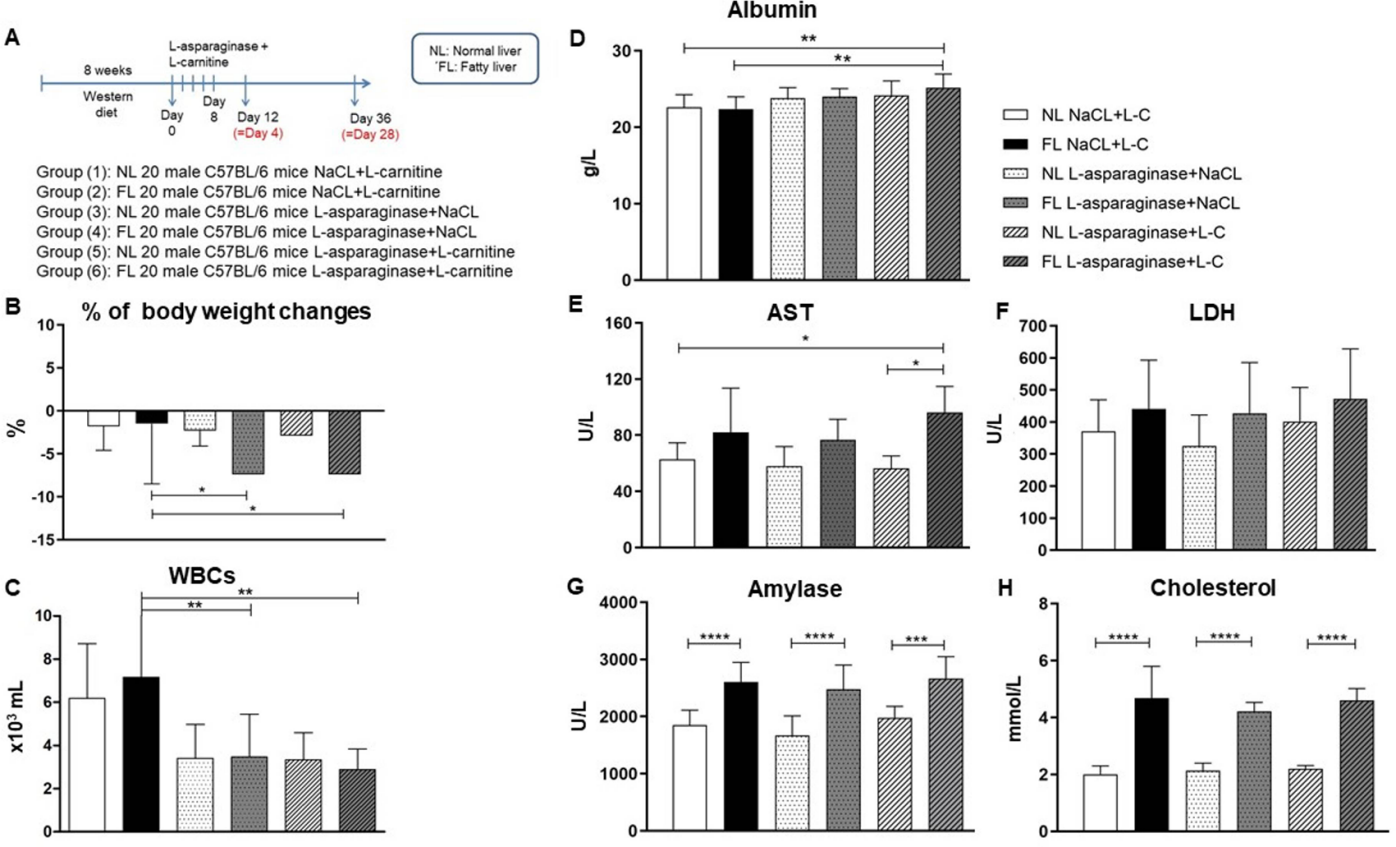
Experimental design (A), % bodyweight changes (B), WBC (C), Albumin (D), AST (E), LDH (F), Amylase (G), Cholesterol (H) shown as means±SD at the 4-day and 28-day time point. * *p* < 0.05, ** *p* < 0.01, *** *p* < 0.001, **** *p*< 0.0001.

Although the dose-escalation study with Oncaspar in female C57BL/6 mice showed already signs of hepatotoxicity in normal and Western-diet fed mice, we decided to establish a second more stringent model to induce a higher degree of hepatotoxicity to better discriminate between the fatty liver and normal liver group and the chemotherapeutic treated and control groups. Based on prior described observation and published data, we choose to increase the feeding time (8 instead of 4 weeks) and to use male instead of female mice. In addition, we tested L-asparaginase, a compound commonly used for the treatment of ALL and known to induce liver toxicity accompanied by steatosis, especially in elderly patients. In order to ameliorate the hepatotoxic effect of L-asparaginase, groups of L-carnitine treatment were added ( Figure 3A). As shown in Figure 3B, bodyweight changes at day 4 after the last treatment indicated that L-C and L-asparaginase treatment resulted in a bodyweight loss which was significantly higher after L-asparaginase administration in FL groups. Interestingly, L-C was not able to ameliorate the effect of L-asparaginase in reducing bodyweight in FL animal groups. Efficiency of L-asparaginase to reduce WBCs is demonstrated in Figure 3C showing no impact of the L-C treatment.

Unlike Oncaspar, the same dosage of L-asparaginase did not decrease ALB levels in NL and FL animal groups at the 4-day time point. Even a significant increase in ALB levels is observed in animals with a fatty liver and L-asparaginase+L-C treatment ( Figure 3D). AST measurements exhibited a slight elevation in FL animal group, which was significant in the L-asparaginase+L-C group compared to the NL control groups ( Figure 3E). LDH enzyme levels were not significantly different between the L-asparaginase or L-C treatment groups. However, a consistent increase is observed due to the Western-diet ( Figure 3F). The amylase measurement showed a highly significant increase in the FL groups independent of the L-asparaginase or L-C treatment ( Figure 3G). Similar results were obtained for cholesterol levels ( Figure 3H). Further analysis of clinical chemistry parameters at the 4-day time point are given in Table 2.

**Table 2.** Results of clinical chemistry measurements at day 4 in sera of male mice treated with L-asparaginase.

Parameters	NL NaCL+L-C	FL NaCL+L-C	NL L-asparaginase + NaCL	FL L-asparaginase + NaCL	NL L-asparaginase + L-C	FL L-asparaginase + L-C
**Markers of liver toxicity:**						
**ALT (U/L)**	48.13±8.03	52.75±13.49	49.33±12.00	58.11±21.20	50.13±11.87	119.63±93.08
**ALP (U/L)**	90.80±11.12	102.11±26.22	88.20±5.33	93.75±9.55	97.50±9.14	97.40±15.12
**TBIL (mmol/L)**	6.80±1.87	5.89±3.14	6.90±0.88	7.20±2.57	7.10±1.10	6.70±2.71
**TP (g/dL)**	4.89±0.25	5.01±0.36	5.02±0.24	5.23±0.18	5.09±0.28	5.36±0.26
**Markers of kidney toxicity:**						
**CK (U/L)**	199.50±128.71	105.50±47.62	202.67±143.76	127.50±60.09	201.00± 130.10	167.63±81.27
**BUN (mmol/L)**	6.65±0.87	5.77±0.81	6.87±0.81	5.55±0.80 ^++^	7.58±0.67	5.18±0.45 ^xxxx^
**CREA (mmol/L)**	19.60±3.37	16.44±1.88 ^*^	17.20±1.55	17.30±1.25	18.60±1.58	15.10±0.74 ^xx^
**Markers of pancreatic toxicity:**						
**Lipase (U/L)**	831.44±377.27	831.67±95.66	730.10±180.98	798.00±126.45	797.30±99.78	909.78±75.94
**Other serum parameters:**						
**Glucose (mmol/L)**	11.56±3.89	11.72±2.21	10.46±2.44	10.64±2.04	11.26±2.13	11.83±3.22
**Triglycerides (mmol/L)**	0.96±0.36	0.97±0.29	0.84±0.29	1.02±0.17	1.11±0.22	0.98±0.21

Alanine Aminotransferase (ALT), Alkaline Phosphatase (ALP), Total bilirubin (TBIL), Total protein (TP), Creatine kinase (CK), Blood urea nitrogen (BUN), Creatinine (CREA). ^*^ Significant to NL NaCl+L-C. ^**+**^ Significant to NL L-asparaginase +NaCl. ^x^ Significant to NL L-asparaginase +L-C. L-C= L-carnitine. NL= normal liver, FL= fatty liver. Means±SD. ^*^, ^**+**^, ^x^ p < 0.05, ^**^, ^++^, ^xx^ p < 0.01, ^****^, ^++++^, ^xxxx^ p < 0.0001.

For the detection of possible late sequestration and/or reversible effects, further animals were analyzed on day 28 after the last application of L-asparaginase. Here, WBCs levels went back to normal and no further changes or reversible effects in serum parameters are found ( Table 3).

**Table 3.** Bodyweights, hematological and clinical chemistry parameters at day 28 in male mice treated with L-asparaginase.

Parameters	NL NaCL+L-C	FL NaCL+L-C	NL L-asparaginase + NaCL	FL L-asparaginase + NaCL	NL L-asparaginase + L-C	FL L-asparaginase + L-C
**% of body weight changes:**	4.0±1.4	11.10±2.7 ^*^	4.00±2.00	10.40±3.50 ^+^	3.00±3.20	8.50±4.70 ^x^
**WBCs:**	6.58±2.28	8.98±3.95	8.32±3.00	8.16±3.19	7.17±1.61	4.95±2.23 ^†^
**Markers of liver toxicity:**						
ALT (U/L)	49.00±6.82	81.50±28.24	43.44±5.73	74.00±31.66	48.67±11.52	72.00±41.31
AST (U/L)	71.86±27.05	107.67±45.95	56.14±8.61	92.20±15.22	74.22±36.73	95.00±46.14
ALP (U/L)	82.38±8.14	104.22±58.67	84.78±8.91	106.75±8.97	93.11±9.99	107.00±32.89
LDH ((U/L)	279.00±37.40	445.60±148.50	278.86±65.03	399.80±92.09	340.38±122.76	347.00±138.80
TBIL (mmol/L)	7.25±1.04	7.44±3.28	6.44±0.73	6.75±1.49	6.44±0.88	6.44±3.00
ALB (g/L)	24.63±0.74	23.89±2.15	23.89±1.27	24.00±1.77	24.44±1.13	24.44±1.74
TP (g/dL)	4.83±0.10	5.02±0.27	4.76±0.13	4.95±0.23	4.86±0.19	5.04±0.27
**Markers of kidney toxicity:**						
CK (U/L)	201.20±120.36	186.60(213.10)	204.29±124.36	69.80±22.04	170.25(171.86)	105.80±90.94
BUN (mmol/L)	6.81±0.67	5.78±0.52	6.98±0.70	5.06±0.32 ^+++^	7.52±0.86	6.16±1.52 ^x^
CREA (mmol/L)	16.95±0.89	17.67±1.50	17.44±1.59	17.25±0.71	18.22±0.97	17.00±1.80
**Markers of pancreatic toxicity:**						
Lipase (U/L)	829.38±91.47	908.00±91.39	849.56±48.78	996.88±407.59	870.00±46.72	911.67±115.20
Amylase (U/L)	2049.50±262.94	2719.00±310.88 ^***^	1967.22±67.46	2814.88±473.88 ^++++^	2096.89±234.27	2626.50±463.02 ^xx^
**Other serum parameters:**						
Glucose (mmol/L)	12.16±2.38	12.66±3.51	12.30±2.71	15.19±2.96	10.01±2.48	11.91±1.64
Triglycerides (mmol/L)	1.27±.022	1.19±0.42	0.94±0.19	0.99±0.35	1.08±0.35	0.92±0.23
Cholesterol (mmol/L)	2.24±0.09	4.48±1.26 ^****^	2.22±0.14	4.93±1.10 ^++++^	2.12±0.29	4.50±1.10 ^xxxx^

Alanine Aminotransferase (ALT), Aspartate Aminotransferase (AST), Alkaline Phosphatase (ALP), Lactate Dehydrogenase (LDH), Total bilirubin (TBIL), Albumin (ALB), Total protein (TP), Creatine kinase (CK), Blood urea nitrogen (BUN), Creatinine (CREA). ^*^ Significant to NL+NaCl. ^†^ Significant to FL+NaCl. ^+^ Significant to NL L-asparaginase+NaCl. ^x^ Significant to NL L-asparaginase+L-C. L-C= L-carnitine. NL= normal liver, FL= fatty liver. Means±SD. ^*^, ^**†**^, ^**+**^, ^x^ p < 0.05, ^**^, ^**††**^, ^++^, ^xx^ p < 0.01, ^***^, ^†††^ p < 0.001, ^****^, ^††††^, ^xxxx^ p < 0.0001.

**Figure 4.**
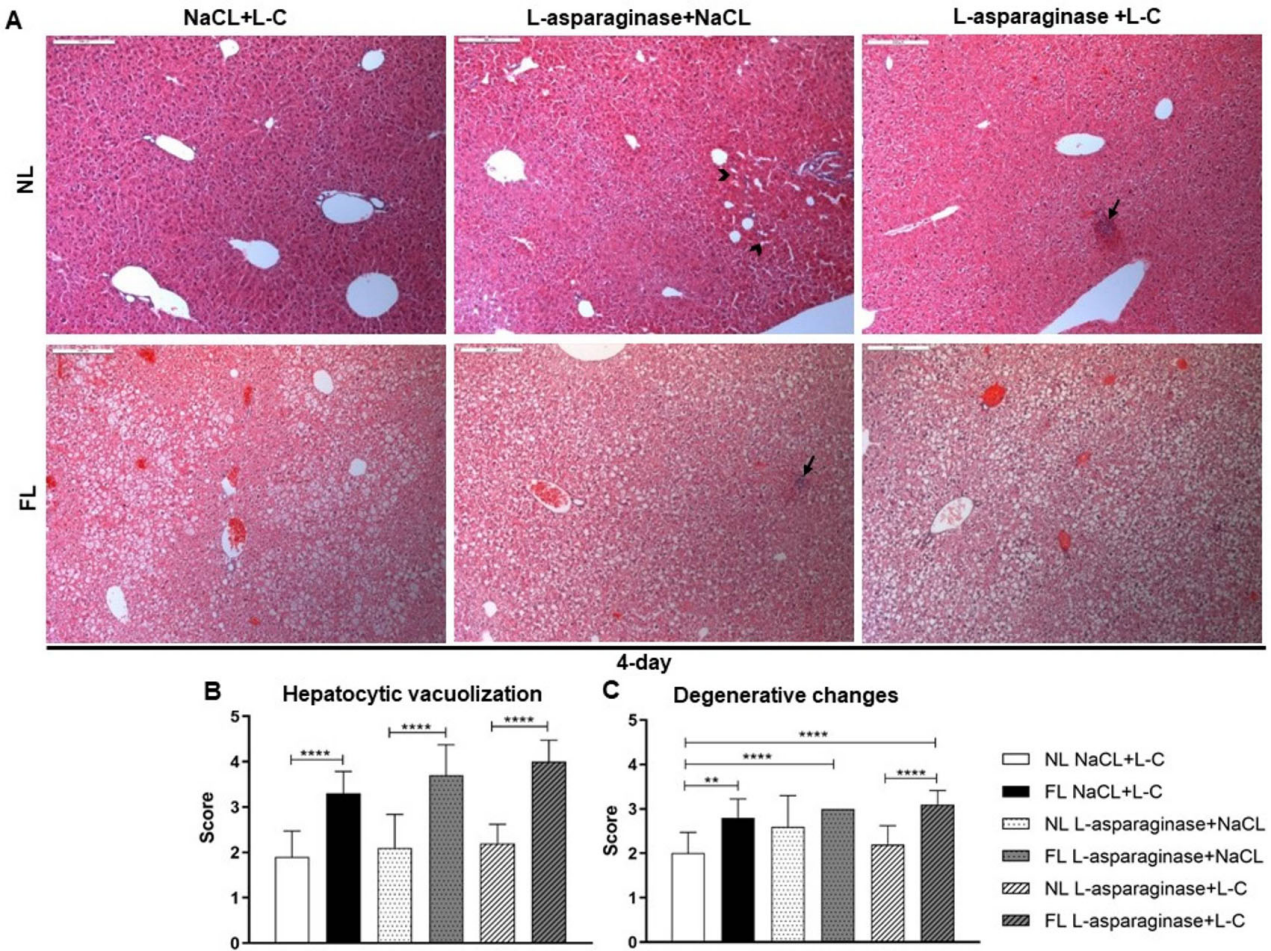
Representative pictures of H&E-stained liver sections at the 4-day time point (100×). Arrows refer to focal area of necrosis. Arrow heads indicate post necrotic area (A). Hepatocytic vacuolization (B), degenerative changes (C) were evaluated and depicted as means±SD. * *p* < 0.05, ** *p* < 0.01, *** *p* < 0.001, **** *p*< 0.0001.

**Figure 5.**
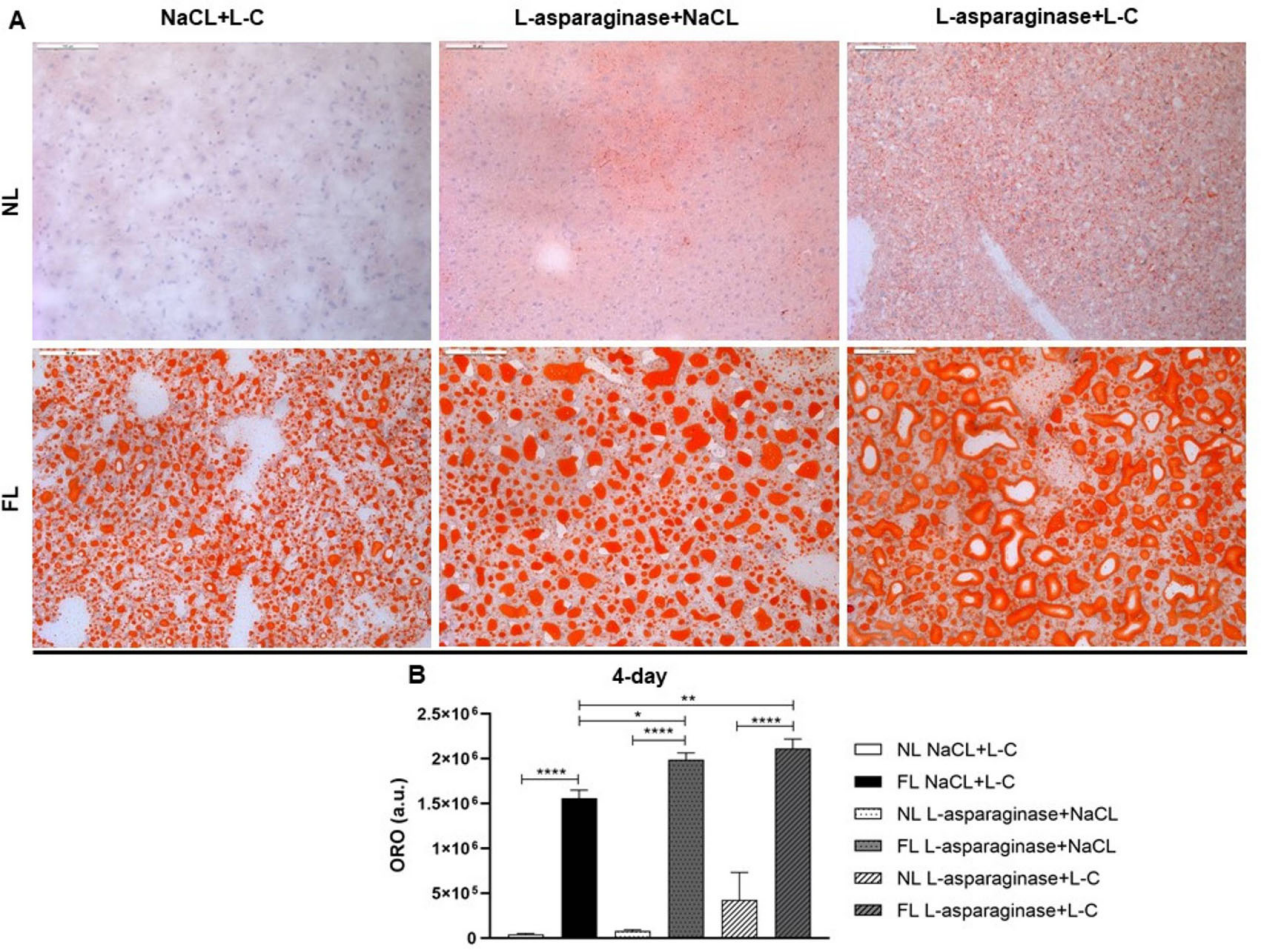
Oil Red O (ORO) of liver sections at the 4-day time point (200×) are depicted (A). Amount of lipids and arbitrary units (a.u.) were measured and shown as means±SD (B). * *p* < 0.05, ** *p* < 0.01, *** *p* < 0.001, **** *p*< 0.0001.

Histopathological analysis revealed a significant increase in hepatocytic vacuolization and in degenerative changes in all FL groups ( Figure 4B/C) at the 4-day time point. Furthermore, FL animals displayed more severe degenerative changes in hepatocytes after L-asparaginase and L-C (FL L-asparaginase+L-C) treatment compared to normal livers from the NL L-asparaginase+L-C treatment group ( Figure 4C), indicating that Western-diet deteriorates the effect of L-asparaginase on hepatocytes. In addition, focal areas of necrosis were observed in liver tissue sections of L-asparaginase treated animals independent of the L-C treatment in all groups. Lipid accumulation evaluated in the ORO staining showed significant increase in all FL animal groups and was further increased by the L-asparaginase treatment ( Figure 5).

**Figure 6.**
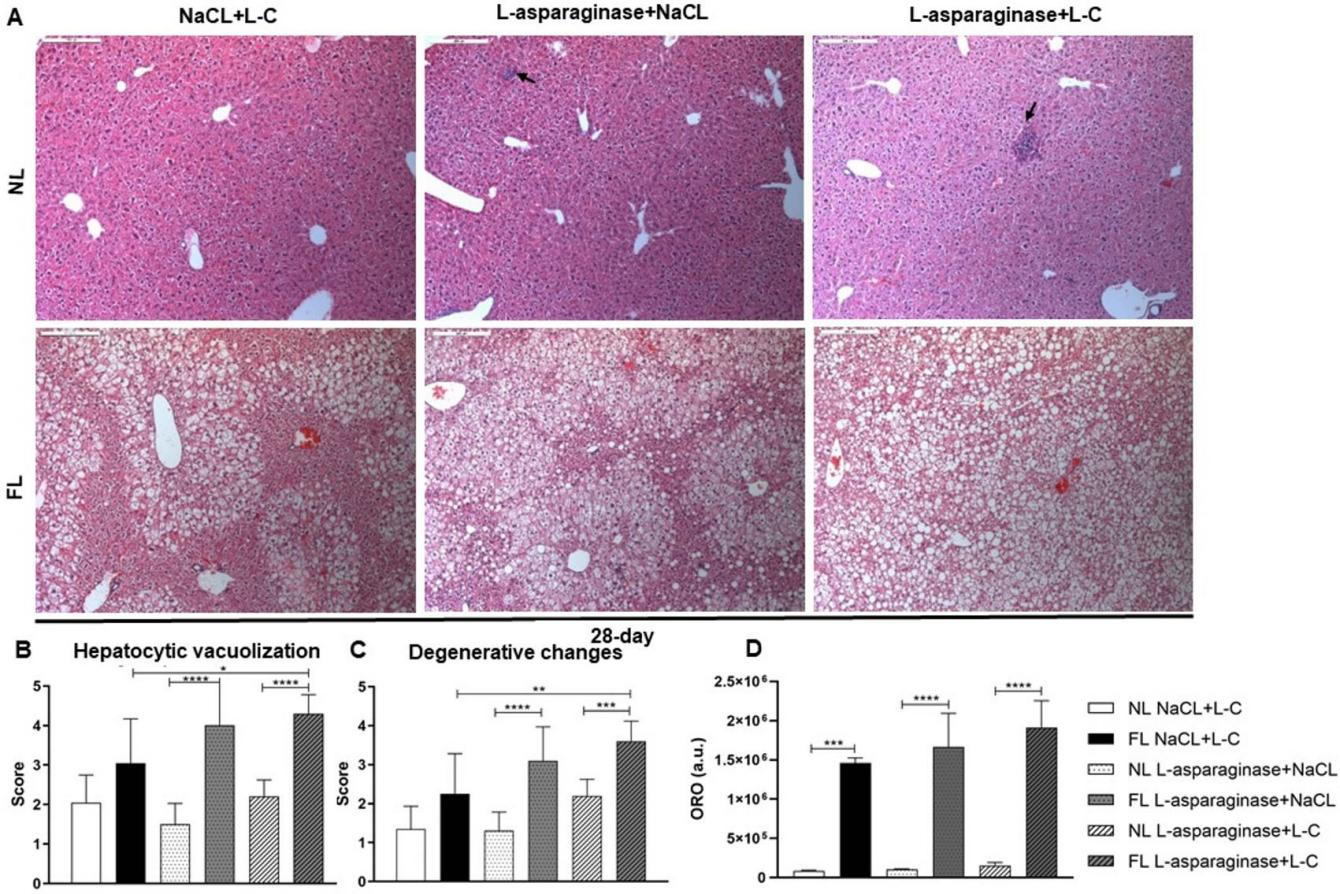
Representative pictures of H&E-stained liver sections at the 28-day time point (100×). Arrows refer to focal area of necrosis (A). Hepatocytic vacuolization (B) and degenerative changes (C) were evaluated and shown as means±SD. * *p* < 0.05, ** *p* < 0.01, *** *p* < 0.001, **** *p* < 0.0001.

Longer feeding with Western-diet resulted in main aggregation of vacuolization in midzonal and centrilobular areas of the liver as depicted in tissue sections at the 28-day time point ( Figure 6). Again, differences in hepatocytic vacuolization, degenerative changes and lipid accumulation were even more prominent between livers of the NL and FL groups and worsened after L-asparaginase and L-C treatment ( Figure 6B-D).

## Discussion

Even though asparaginases are efficiently used as chemotherapeutic drugs side effects especially in elderly patients with pre-existing conditions can counteract their unrestricted use. ^5^ It has been shown that non-healthy livers are more susceptible to chemotherapy-induced adverse effects ^15^^,^^16^ and that chemotherapeutic drugs cause liver toxicity accompanied by steatosis especially in older patients. ^5^ Therefore, we aimed to develop a fatty liver (steatotic) model mimicking the situation in humans to study the effect of asparaginases induced liver toxicity in C57BL/6 mice to test L-carnitine as a protective agent to ameliorate asparaginase-induced liver toxicity.

Bodyweight changes, blood parameters and clinical chemistry as well as histopathological changes in the livers of mice were analyzed and efficacy of the chemotherapeutic drug was verified by counting the WBCs. Asparaginases were reported to cause reduction in bodyweight ^17^ due to increased catabolism, ^18^ which could be also verified in our models in all treatment groups. As expected, the WBCs count showed a marked reduction after administration of Oncaspar or L-asparaginase in all treatment groups. ^19^

Clinical chemistry parameter measurement showed a significant decline in albumin and total protein levels after Oncaspar administration, independent of the condition of the liver by inhibition of protein synthesis associated with the main mechanism of the drug. ^20^ Hypoalbuminemia is one of the major abnormalities associated with Oncaspar treatment ^21^ with a consequent hepatic toxicity. In contrary, treatment of L-asparaginase resulted in a significant elevation of albumin in FL L-asparaginase+L-C group. This might be attributed to the fact that L-asparaginase is known to have a shorter half-life than Oncaspar ^22^ that consequently enhances clearance rate in the circulatory system via native proteases. ^23^

ALT and AST levels in serum indicating liver toxicity, showed only non-significant slight elevations, mainly due to the Western-diet. Our results were in line with previously published data showing no signs of hepatotoxicity after treating mice with Oncaspar. ^17^

Amylase, as a marker of pancreatic toxicity, showed a significant increase in FL animals treated with Oncaspar especially in the highest dosage group. However, the model using L-asparaginase showed only amylase elevation due to the Western-diet suggesting that feeding animals with high fat diet stimulate the secretion of amylase as previously described. ^24^ Several investigations argued whether asparaginases attributed to hypercholesterolemia ^25^ or hypocholesterolemia. ^26^ Our models demonstrated a significant increase of cholesterol in animals fed with Western-diet and has been expected and reported in another study. ^27^ The administration of Oncaspar seemed to reduce this increase, while L-asparaginase did not show any effect of the Western-diet-induced cholesterol elevation.

Histopathological analysis of H&E-stained liver tissue sections demonstrated hepatocytic degenerative changes, like vacuolization, as a main finding of hepatotoxicity associated with Oncaspar administration but also with Western-diet alone ( Figure 2). Hepatic changes reached to a moderate severity grade and degenerative changes were observed in a dose-dependent manner after Oncaspar treatment in NL and FL groups showing a deteriorating effect in the FL groups. These results confirm former investigations in an *ex vivo* model, in which a higher liver toxicity in histopathological analysis induced by L-asparaginase treatment in fatty liver was reported. ^28^

The second model using L-asparaginase histopathological analysis revealed that the observed significant increase of hepatic vacuolization was mainly due to the Western-diet. In regard to the degenerative changes, animals of the NL groups treated with L-asparaginase showed also mild changes and scored higher in the FL groups.

ORO-stained liver sections showed a significant accumulation of lipids due to the Western-diet and the administration of Oncaspar, at least in the highest dosage group. Thus, Oncaspar treatment deteriorated the effect of Western-diet in accumulating lipids and our model showed a clear sign of hepatosteatosis. L-asparaginase treatment of Western-diet fed animals showed hereby similar results ( Figure 5). As already described asparaginases induced hepatotoxicity is attributed to inhibition of protein synthesis and related to mitochondrial dysfunction ^8^ and impairment of energy production leading to micro-vesicular steatosis, hepatocytes necrosis or apoptosis. ^29^ Lipoproteins and lipids get exported and accumulate in hepatocytes resulting in steatosis and liver dysfunction. ^30^

Vacuolization mainly aggregated in midzonal and centrilobular areas of liver tissues. We noticed that more severe vacuolization of hepatocytes depicted at the 28-day time point in FL animal groups was a result of longer feeding with Western-diet. We could demonstrate the impact of Oncaspar and/or L-asparaginase treatment in worsening the effect of Western-diet in accumulating lipids in hepatocytes and thus our model showed a clear sign of hepatosteatosis. Our findings were in compliance with former studies in which accumulation of lipids in hepatocytes after feeding mice with high fat content diet was observed resulting in hepatic steatosis. ^31^

To improve the animal model in order to discriminate better between the effects of Western-diet and chemotherapy, the feeding time was increased from 4 weeks to 8 weeks and male mice, which are described as more vulnerable for the induction of fatty liver, ^32^ were used. Male mice develop rather symptoms of human metabolic syndrome than females, ^33^ due to estrogen which provide a protective capacity against non-alcoholic fatty liver disease. ^34^ For these reasons, we substituted females with males in our experiment to develop an animal model showing metabolic syndrome mimicking those of overweight humans.

Due to the antioxidant properties of L-carnitine it has been utilized as a protective agent against toxicity that might be triggered by cisplatin or doxorubicin as anti-cancer drugs. ^35^ In our model, L-C was not able to ameliorate the effect of L-asparaginase on bodyweight changes in FL animal groups. As already described it also had no effect on the efficacy of L-asparaginase in reducing WBC’s in our model. ^17^ However, L-C administration resulted in a non-significant increase in our tested clinical chemistry parameters. Histopathological findings did not show significant improvement based on the evaluation of hepatocytic vacuolization. On the contrary, degenerative changes was even deteriorated after L-C application in FL animal groups after asparaginase treatment. Our findings were in line with previous studies that proved ineffectiveness of L-C in ameliorating parameters referring to hepatic dysfunction as well as impairing hepatic steatosis histologically in a C57BL/6 mouse model. ^36^

In summary, our established models showed mild toxic effects of Oncaspar with slight to moderate liver toxicity. In Western-diet-fed animals, a higher degree of hepatocyte damage and vacuolization could be observed in liver tissue compared standard diet fed animals. Only mild signs of liver toxicity could be observed after using L-asparaginase, while more (but not significant) severe signs, were detected in animals with fatty livers. L-C could not ameliorate the induced toxicity and did not show an impairment of the efficacy of Oncaspar or L-asparaginase. However, with the mild severity grade of the liver toxicity model it might be not severe enough to discriminate between effects induced by the Western-diet and chemotherapeutic drug in order to subsequently test potential ameliorating drugs.

## Data availability

### Underlying data

Figshare: Underlying data for ‘Effects of asparaginases and L-carnitine on Western-diet-induced hepatosteatosis in mice’

Measured parameters of dose-escalation study of Oncaspar. https://doi.org/10.6084/m9.figshare.17032946. ^37^

Measured parameters of L-asparaginase study at the day 4 time point. https://doi.org/10.6084/m9.figshare.17032391. ^38^

Measured parameters of L-asparaginase at the 28 days’ time point. https://doi.org/10.6084/m9.figshare.17068124. ^39^

Results of histopathological analysis. https://doi.org/10.6084/m9.figshare.17068148. ^40^

### Reporting guidelines

Figshare: ARRIVE guideline checklist for ‘Effects of asparaginases and L-carnitine on Western-diet-induced hepatosteatosis in mice https://doi.org/10.6084/m9.figshare.18620330. ^41^

Data are available under the terms of the Creative Commons Zero “No rights reserved” data waiver (CC0 1.0 Public domain dedication).
