# Supplementary material for: Effects of asparaginases and L-carnitine on Western-diet-induced hepatosteatosis in mice
Source: F1000Res. 2022 Feb 1;11:128. [Version 1] doi: 10.12688/f1000research.75870.1 (PMC10366552; doi:10.12688/f1000research.75870.1)
Supplement: Peer review history

# Peer review: Reviewer response for version 1

**DOI:** 10.5256/f1000research.79799.r182977

**Published:** 2023-07-24

**Authors:** Olamide B Adelusi

**Affiliations:** 1 Department of Pharmacology, Toxicology and Therapeutics, University of Kansas Medical Center, Kansas City, KS, USA

In this study, the authors aimed to investigate the hepatotoxicity of two asparaginases in normal and western diet fed mice, as well as L-carnitine as a potential antidote to asparaginase induced hepatotoxicity. The work is interesting with potential clinical relevance, however, several points should be addressed:

- The methods section is missing some details such as when L-carnitine was given and the route of administration of L-asparaginase.
- How were the doses of Oncaspar, L-asparaginase and L-carnitine arrived at? Are these human relevant doses? An explanation would be helpful.
- How was the number of animals for the different experiments decided? For the Oncaspar study, 6 mice per group were used while for L-asparaginase a much larger number (20 mice per group). Is one of the experiments statistically insufficiently powered or overpowered?
- Why were some experiments performed using Oncaspar and others using L-asparaginase?
- Is the western diet model reiterative of what is observed clinically? Why not use aged mice since the elderly are the group most at risk of asparaginase toxicity in clinical settings?
- Why was 4 days after the treatments selected for endpoint measurement? How long after the treatments would the effects of Oncaspar and L-asparaginase be expected to persist? It is possible that the liver has started to recover from the insult in the days since the last treatment. Perhaps measuring liver injury endpoints closer to the end of treatment might show more pronounced effects.
- To the previous point, the effect of Oncaspar and L-asparaginase with or without L-carnitine on liver regeneration should be investigated. Immunostaining for cell cycle proteins such as PCNA or Ki67 is one way to do this.
- Liver to body weight ratio would be a more relevant parameter in this case than gross body weight.
- Many graphs are missing error bars e.g. figure 1C, 2B, 2C, etc. Also, the number of mice per group should be indicated in the figure legends.
- Some of the measurements such as renal and pancreatic injury parameters can be removed or moved to supplements as they do not contribute to the narrative.
- In figure 2, the oil red o images should be included.
- In figure 3, it is not clear if the results are from day 4 or day 28.
- While the authors showed some clinical and histological changes with Oncaspar and L-asparaginase treatment, these are very small in magnitude and may not be clinically significant. Some discussion of what is observed in patients is necessary to contextualize these findings.
- Given the relatively small increase in steatosis and liver injury in FL + L-asparaginase group compared to FL + NaCl, it is difficult to tease out any protective effect of L-carnitine. A more robust toxicity phenotype is necessary to give conclusive evidence on the effect of L-carnitine on L-asparaginase induced hepatotoxicity.

Is the work clearly and accurately presented and does it cite the current literature?

Partly

If applicable, is the statistical analysis and its interpretation appropriate?

Partly

Are all the source data underlying the results available to ensure full reproducibility?

Yes

Is the study design appropriate and is the work technically sound?

Partly

Are the conclusions drawn adequately supported by the results?

Partly

Are sufficient details of methods and analysis provided to allow replication by others?

Partly

Reviewer Expertise:

Liver toxicology, acetaminophen induced liver injury, liver regeneration

I confirm that I have read this submission and believe that I have an appropriate level of expertise to confirm that it is of an acceptable scientific standard, however I have significant reservations, as outlined above.

# Peer review: Reviewer response for version 1

**DOI:** 10.5256/f1000research.79799.r161777

**Published:** 2023-07-05

**Authors:** Ruchi Bansal

**Affiliations:** 1 Translational Liver Research, Department of Medical Cell Biophysics, Technical Medical Center, Faculty of Science and Technology, University of Twente, Enschede, The Netherlands

This is an interesting and relevant study where the authors have studied the effects of Asparaginases and L-carnitine on western diet-induced hepatosteatosis. The authors have demonstrated asparaginase-induced hepatotoxic effects and that L-carnitine did not show any protective effect on the toxicity of asparaginases. I have several suggestions and questions which if implemented can improve the quality of the work.

 1. In the introduction, Spectrila could be included as a recombinant "native L-asparaginase isolated from *Escherichia coli" *similar to Oncaspar.

 2. Experimental design:

- The animal study experimental design should be improved. The diet composition and the administration route (e.g., not available for Spectrila), etc. should be provided. When L-C (300 mg/kg body weight) was injected (before or after Spectrila) is not clear.
- It is unclear why the authors used young animals in this study. I would have expected the use of older animals to mimic more elderly age groups.
- In the first study, different asparaginase was investigated as compared to the second animal study. Is this first short-term study used to guide the second animal study? If yes, then why did the the authors change female mice to male mice? 
- In the first animal study, only 5 mice are used while in the second animal study, 10 mice per group are used. Why is that the case? 
- In my opinion, the choice of model should be motivated by the clinical situation. Is there a way to justify their use of animal models motivated by the clinical situation?

3. Results:

- In my opinion, having results split into figures and tables is confusing. I propose to include the clinical chemistry measurement results in the table and other results i.e., body weight, WBCs, staining, etc. in the figure.  
- In the first animal study, I would have expected concentration-dependent effects, however the effects are not concentration-dependent. Why this is the case? Can the authors explain these observations?
- Some of the error bars are missing in Figure 1?
- Body weight changes presented from day 0 or from the day of the start of the feeding time? 
- Is the change in body weight reflected in food consumption? Why did the animals lose weight? Is the organ weight data available?
- Oil-red-o staining results should be included as supplementary or in Figure 2. Error bars are missing for some of the bars. 
- It is not clear what the authors mean by degenerative changes and how are they assessed. 
- In Figure 3, it is not clear if the data is presented from a 4-day or 28-day time point. this should be clarified. Also, the n number should be mentioned in all the figures and table legends. 
- It is surprising that the control (healthy and untreated/vehicle-treated FL) groups are missing but the authors mentioned under table 3 that the significance was calculated versus the FL+NaCl group while it is not mentioned in the experimental design in Figure 3A. These groups should be included for a fair comparison. 
- Please see my comments for the first animal study related to body weights and organ weights. 

In general, it would have been fair to compare in one study (containing male and female mice) the effect of recombinant L-asparaginase (Spectrila) and pegaspargase (Oncaspar) with and without L-Carnithine. It is not entirely clear why the studies were performed as they were performed here. 

 4. Discussion: In the discussion section, it is good to motivate the study design i.e., the choice of the model, the choice of different asparaginases, the choice of male versus female, doses, and administration route with the clinical situation or treatment regimen. Also if the pre-existing conditions such as hepatopathies are comparable and if the toxic effects of asparaginases are comparable to what is observed in clinical settings. Finally, how this study can improve the clinical trials and/or could be useful should be elaborated.

Is the work clearly and accurately presented and does it cite the current literature?

Yes

If applicable, is the statistical analysis and its interpretation appropriate?

Partly

Are all the source data underlying the results available to ensure full reproducibility?

Partly

Is the study design appropriate and is the work technically sound?

Partly

Are the conclusions drawn adequately supported by the results?

Partly

Are sufficient details of methods and analysis provided to allow replication by others?

Partly

Reviewer Expertise:

Fatty liver diseases, animal models, diagnosis and therapeutics, single-cell omics, hepatic steatosis, liver inflammation, liver fibrosis.

I confirm that I have read this submission and believe that I have an appropriate level of expertise to confirm that it is of an acceptable scientific standard, however I have significant reservations, as outlined above.

# Peer review: Reviewer response for version 1

**DOI:** 10.5256/f1000research.79799.r121811

**Published:** 2022-02-07

**Authors:** Matías A. Avila

**Affiliations:** 1 Hepatology Program, CIMA, University of Navarra, Pamplona, Spain

In this study Mahmoud Assar and coworkers evaluate the hepatotoxicity of two asparaginases formulations in mouse models of fatty liver disease. This is a meaningful study, given the use of asparaginases for the treatment of acute lymphoblastic leukemia (ALL) and the increased liver toxicity found in the clinical setting when patients with hepatopathies, or elderly individuals are treated. Fatty liver develops with age in the general population, therefore the chosen mouse model of fatty liver disease is very relevant. The authors also test the potential protective effects of L-carnitine. The major findings indicate enhanced asparaginase toxicity in mice with fatty liver, mainly for the more stable asparaginase formulation Oncaspar. L-carnitine showed no protective effect. This is a relevant work providing useful information with clinical relevance. I only have a few suggestions for the authors that if addressed could improve their report.

1. It would be helpful to justify in the Methods section how the doses and administration regimes of Oncaspar and L-asparaginase (Spectrila) to mice were selected.
2. The composition of the Western diet should be detailed.
3. Were there signs of inflammation in the livers of mice fed this Western diet? If so, did L-asparaginases administration have an effect on inflammatory parameters?
4. Could you please discuss why Oncaspar seemed to reduce cholesterol levels increased by Western diet feeding?
5. Can you please add a short comment on the potential relevance of the major findings of this study regarding the application of L-asparaginase formulations in patients with ALL.
6. Please provide information about the size of space bars that are given in the different figures in the legends to the figures.
7. Can you provide a list of abbreviations (NL, NL L, FL L) because this would increase the readability of the paper.
8. Please change “NaCL” for NaCl in all figures.

Is the work clearly and accurately presented and does it cite the current literature?

Yes

If applicable, is the statistical analysis and its interpretation appropriate?

Yes

Are all the source data underlying the results available to ensure full reproducibility?

Yes

Is the study design appropriate and is the work technically sound?

Yes

Are the conclusions drawn adequately supported by the results?

Yes

Are sufficient details of methods and analysis provided to allow replication by others?

Partly

Reviewer Expertise:

Experimental hepatology, liver injury/regeneration, liver fibrosis, carcinogenesis, metabolism, gene expression, epigenetics.

I confirm that I have read this submission and believe that I have an appropriate level of expertise to confirm that it is of an acceptable scientific standard.
